# The Optimization of Hole Injection Layer in Organic Light-Emitting Diodes

**DOI:** 10.3390/nano14020161

**Published:** 2024-01-11

**Authors:** Xiaolin Xing, Ziye Wu, Yingying Sun, Yunlong Liu, Xiaochen Dong, Shuhong Li, Wenjun Wang

**Affiliations:** 1 School of Physical Science and Information Technology, Liaocheng University, Liaocheng 252059, China; 15269073659@163.com (X.X.); qw2320329838@163.com (Z.W.); 17353609715@163.com (Y.S.); iamxcdong@njupt.edu.cn (X.D.); lishuhong@lcu.edu.cn (S.L.); 2 Shandong Provincial Key Laboratory of Optical Communication Science and Technology, Liaocheng 252059, China; 3 Key Laboratory of Flexible Electronics (KLOFE), Institute of Advanced Materials (IAM), School of Physical and Mathematical Sciences, Nanjing Tech University (NanjingTech), Nanjing 211800, China

**Keywords:** organic light-emitting diodes, hole injection layers, hole mobility, energy level matching, post-treatment methods

## Abstract

Organic light-emitting diodes (OLEDs) are widely recognized as the forefront technology for displays and lighting technology. Now, the global OLED market is nearly mature, driven by the rising demand for superior displays in smartphones. In recent years, numerous strategies have been introduced and demonstrated to optimize the hole injection layer to further enhance the efficiency of OLEDs. In this paper, different methods of optimizing the hole injection layer were elucidated, including using a suitable hole injection material to minimize the hole injection barrier and match the energy level with the emission layer, exploring new preparation methods to optimize the structure of hole injection layer, and so on. Meanwhile, this article can help people to understand the current research progress and the challenges still faced in relation to the hole injection layer in OLEDs, providing future research directions to enhance the properties of OLEDs.

## 1. Introduction

Since the significant discovery that Eastman Kodak launched commercial organic light-emitting diodes (OLEDs) with a two-layer structure in 1987, the exploration of OLEDs has begun and increased. In recent years, OLEDs have become the most popular future display and lighting technology due to their superior characteristics, such as higher visual experiences, lower power consumption, design flexibility, and so on [1]. Driven by the rising demand for superior displays in smartphones, the global OLED market is nearly mature now. However, OLEDs have also faced various challenges, such as shorter lifetime, lower efficiency with severe efficiency roll-off, etc. The development of outdoor lighting with OLEDs was impeded by the device degradation from exposure to ultraviolet light and excessive moisture.

**Figure 1:**
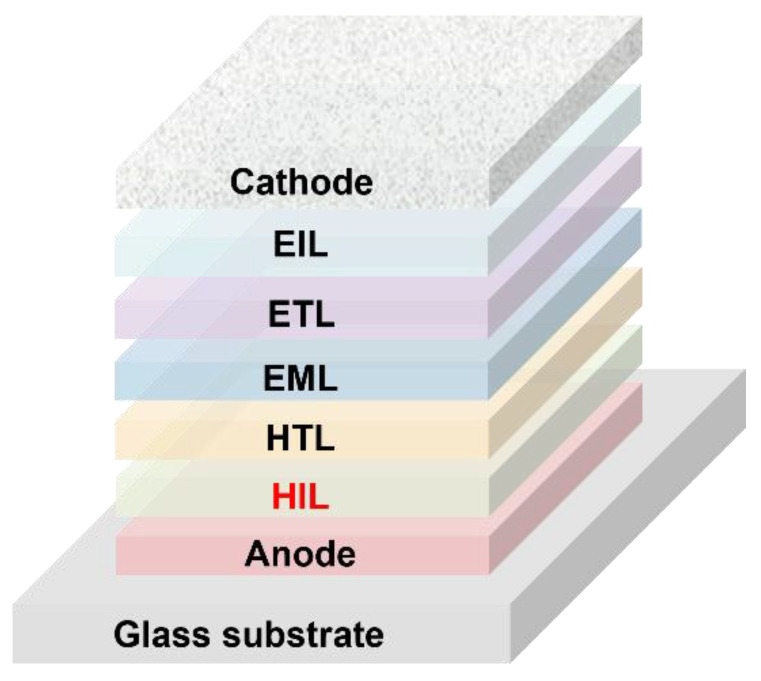
Device structure of a typical OLED.

In order to enhance the performance of OLEDs, various techniques have been developed. One of the most effective strategies is the incorporation of hole injection layers (HILs) between the hole transport layer (HTL) and electrodes. The HIL promotes the charge injection from the electrode to the emissive layer (EML), while preventing excitons from being quenched by the electrodes at the same time. The typical device configuration that has been widely reported includes indium tin oxide (ITO) (anode)/HIL/HTL/EML/electron transport layer (ETL)/electron injection layer (EIL)/aluminum (Al) (cathode) (Figure 1). 

**Figure 2:**
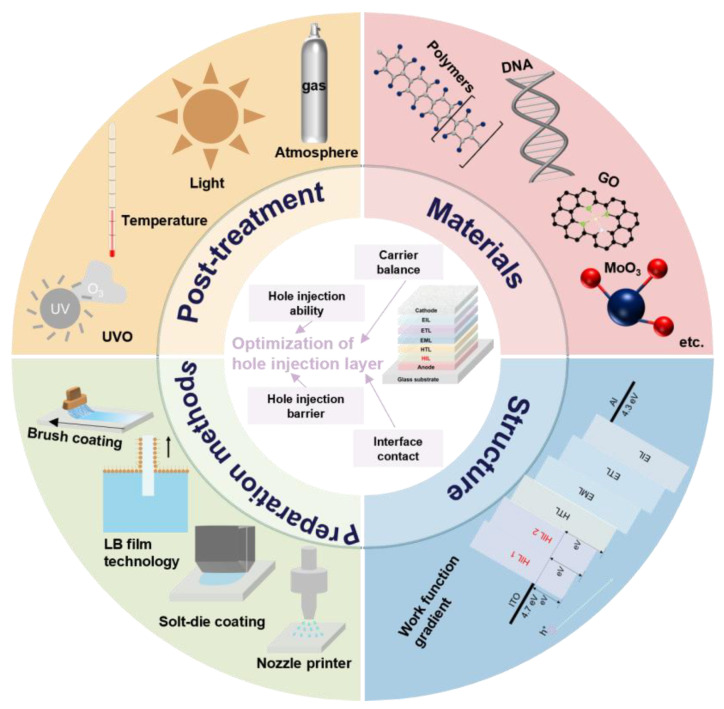
Gist of article.

This paper focuses on the latest progress in improving OLED performance by modifying HIL, mainly in terms of materials, structure, preparation methods, and the influence of post-treatment. We mainly reviewed the critical material strategies that were implemented to achieve high-performance OLEDs using organic or inorganic hole injection materials. Subsequently, the recently reported OLEDs based on different structures of preparation and the effect of post-treatment methods on OLEDs were summarized. Finally, it concluded with a discussion of perspectives and remaining issues for the future. The highlights are summarized in Figure 2.

## 2. Hole Injection Layer Materials

With the development of materials design and synthesis, new-type HILs with different structures and functional groups appeared. An effective hole injection material must be of relatively high glass transition temperature, good hole mobility for high conductivity, and high triplet energy to block triplet exciton. In the quest to avoid charge traps or carrier recombination, energy level alignments and the presence of low interface states are valuable [2,3]. Different materials provide different work functions, hole mobility, and hole injection capabilities. Then, the selection of HIL materials significantly impacts the performance of OLEDs.

To date, a wide range of HIL materials have been developed and extensively employed to enhance the OLED performance. Here, we explored the HIL materials from various categories, including organic materials and inorganic materials.

### 2.1. Organic Materials

**Figure 3:**
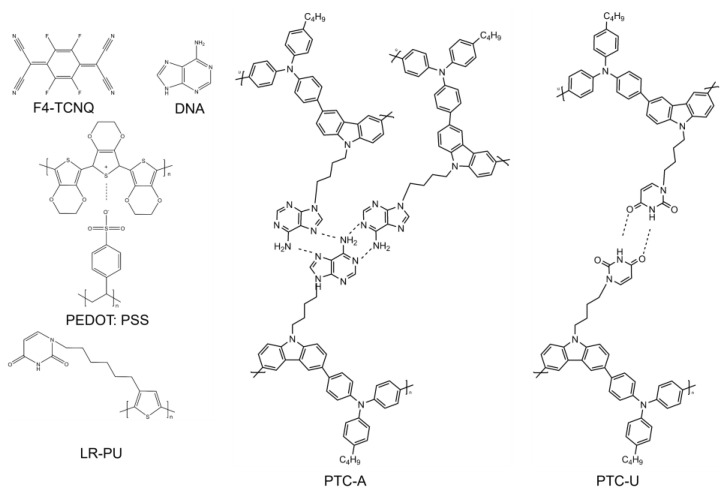
The structures of organic materials used as HILs.

Conductive polymers and organic small molecules are often used as HILs in OLEDs. The requirements for HILs include excellent optical and electrical characteristics, such as high conductivity, transparency, as well as low surface roughness [4,5,6]. In order to optimize the HILs to enhance the OLED performance, the diffusion properties, morphological stability, and environment friendliness of HILs are often considered. Figure 3 presents the structures of organic materials used as HILs.

To reduce the manufacturing cost, solution-processed organic HILs have been implemented to enhance device performance [7,8,9]. Conducting polymers represent a highly promising class of organic semiconductors due to their unique optical and electrical properties. For example, PEDOT:PSS, considered as one of the mostly widespread substances for the preparation of hole injection/transport layers (HITLs) in solution-processed OLEDs, is characterized by suitable energy levels, favorable hole mobility, and an easy ability to form smooth films [10,11]. Amare Benor et al. also proved that carrier injection can be enhanced by adjusting the work function (WF) of PEDOT:PSS, thereby improving the device power efficiency (PE) [10].

However, PEDOT:PSS tends to corrode indium tin oxide (ITO) due to hygroscopicity and strong acidity, which limits the durability and reliability of the devices. Moreover, it has the potential to induce instability and profound deterioration in thin-film optoelectronic devices [2]. Based on the above, Sha Wu et al. introduced a PH-neutral PEDOT:PSS to form double-layer HILs with acidic PEDOT:PSS, which improved the OLED performance [12]. Mingguang Li et al. reduced the acidity of PEDOT:PSS HILs and constructed continuous hole transport channels by using a thermal imprinting and vapor annealing method. Finally, OLEDs with current efficiency (CE) of 36.62 cd A^−1^, PE of 27.60 lm W^−1^, and external quantum efficiency (EQE) of 18.80% were obtained [13].

More materials are also proposed to substitute PEDOT:PSS. For instance, polythienothiophene:poly(perfluoroethylene-perfluoroethersulfonic acid) (PTT:PFFSA), which was used as an HIL in OLEDs, can suppress the accumulation of charges at the anode/HIL interface [14]. Compared to OLEDs featuring PEDOT:PSS as the HIL, the device utilizing PTT:PFFSA as an HIL displays enhanced efficiency, slower luminance decay, and slower increases in operating voltage.

The diffusion properties of the HIL materials will change the properties of HTLs and should be considered. For instance, the tetrafluorotetracyanoquinodimethane (F_4_-TCNQ) used as an HIL with different thicknesses diffuses into the HTL with different lengths and depths and leads to the F_4_-TCNQ ionization and the p-type doping of F_4_-TCNQ with the hole transporting material. The application of an electric field has been found to increase both the diffusion depth and length of F_4_-TCNQ to the HTL. It was found that the diffusion of a 1 nm thickness F_4_-TCNQ layer induced exciton dissociation within the EML and led to a decrease in the stability of OLEDs [15,16].

Moreover, F4-TCNQ has electron affinity and morphological stability [17]. When it is doped into other HILs, it can increase the HOMO level. Xiao-Lan Huang et al. reported the use of a simple small-molecule organic HIL known as N,N,N′,N′-tetra(4-methoxyphenyl)[2,2′-binaphthalene]-6,6′-diamine for OLEDs. When the material doped with F4-TCNQ, the HOMO level increased from 4.68 eV to 5.27 eV, enhancing the hole conductivity up to 2.64 × 10^−4^ S m^−1^ compared to the neat film of 1.68 × 10^−4^ S m^−1^ [18].

**Figure 4:**
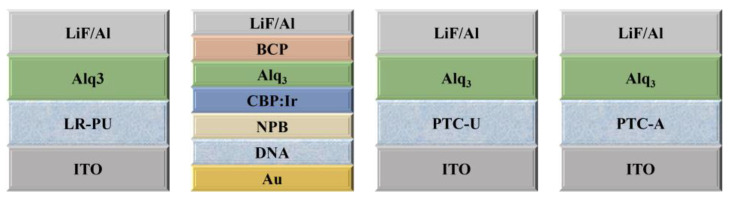
The structures of OLEDs with biomaterial HILs.

Biological materials are non-toxic and environmentally friendly, which was explored as HILs in OLEDs. The nucleic acid bases (nucleobases) present in the DNA biopolymer, which possess a diverse range of energy levels, can be easily thermally evaporated and can be used as HILs without additional modification. The intrinsic physical and chemical affinity between the thin-layer adenine and the gold electrode enhances hole injection, leading to a significant increase in CE and luminance [19]. In 2012, Chih-Chia Cheng et al. found that the physically cross-linked low-regioregular nucleobase functionalized poly(3-thiophene)s (P3HTs) incorporating overhanging uracil groups (LR-PU) could significantly smooth the surface and enhance solvent resistance, thermal stability, hole injection, and electron-blocking ability of films. The prepared OLEDs had a maximum luminance of 6310 cd m^−2^ and a luminescence efficiency of 1.24 cd A^−1^, outperforming the OLEDs with LR-P3HT and HR-P3HT as a mono HIL [20]. In 2015, PTC-U, a DNA-mimetic π-conjugated poly(triphenylamine-carbazole) with pendent uracil groups, was employed as an HIL. After that, Chih-Chia Cheng et al. prepared new poly(triphenylamine-carbazole)-based adenine (PTC-A) as an HIL, which possessed high self-complementarity in both solution and solid states due to the formation of adenine–adenine (A–A) pairs resulting from induced hierarchical self-assembly. And the device performance based on PTC-A had the highest efficiency compared with those utilizing PTC-U and PEDOT:PSS [21,22]. The structures of OLEDs with biomaterial HILs are shown in Figure 4, and the optical properties of the devices with biomaterial HILs are shown in Table 1. 

### 2.2. Inorganic Materials

Even though organic materials reduced the preparation cost, the improvement in OLED efficiency is still limited. Transition metal oxides (TMOs) and two-dimensional (2D) materials are often used inorganic HIL materials. Transition metal oxides (TMOs), like molybde-num trioxide (MoO_3_), tungsten trioxide (WO_3_), and vanadium pentoxide (V_2_O_5_), and metal sulfides like tungsten sisulfides (WS_2_), have been increasingly employed as HILs due to their high technological compatibility, appropriate electronic characterization, and low light absorption in the visible spectrum [23,24].

TMOs have been identified as promising candidates for replacing PEDOT:PSS as HILs for OLEDs [25,26,27,28]. In 1996, Tokito et al. first used vanadium, molybdenum, and ruthenium oxide films to modify the anode, which increased the hole injection and significantly improved the OLED performance [29]. Matsushima et al. implemented 0.75 nm thick MoO_3_ as HILs for OLEDs, and the ohmic hole injection effect formed by the ultrathin MoO_3_ HIL significantly reduced the driving voltage [30]. You et al. reported efficient green fluorescent OLEDs with lower turn-on voltage and excellent stability of over 50,000 h by using the MoO_3_ layer as HILs [31]. Rui Liu et al. prepared a wavelength-tunable multicolor microcavity (μC) OLED device only by controlling the thickness of the MoO_3_ hole injection layer, and maximal brightness of 140,000 cd m^−2^ was obtained [32]. 

The thermally evaporated MoO_3_ has high conductivity and is beneficial to energy alignment because of the oxygen vacancies, which can also be fabricated through a solution method [32,33]. Xiaowen Zhang et al. used solution-processable MoO_x_ HILs to increase the luminous efficiency of OLEDs. It was found that the tailored surface of WF and proper hole injection arose by the oxygen vacancies, optimizing the carrier balance in OLED and leading to enhanced performance [33].

**Figure 5:**
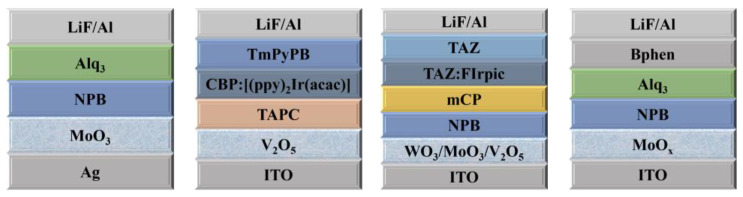
The structures of OLEDs with transition metal oxide HILs.

At the same time, the transmittance and conductivity of TMO also affect the performance of devices. For example, although V_2_O_5_ has the highest WF compared with WO_3_ and MoO_3_, the device performance based on V_2_O_5_ HIL is inferior to the latter due to its high resistance and low transmittance [34]. The structures of OLEDs with transition metal oxide HILs are shown in Figure 5, and the optoelectric properties of OLEDs based on transition metal oxide hole injection materials are shown in Table 2.

**Figure 6:**
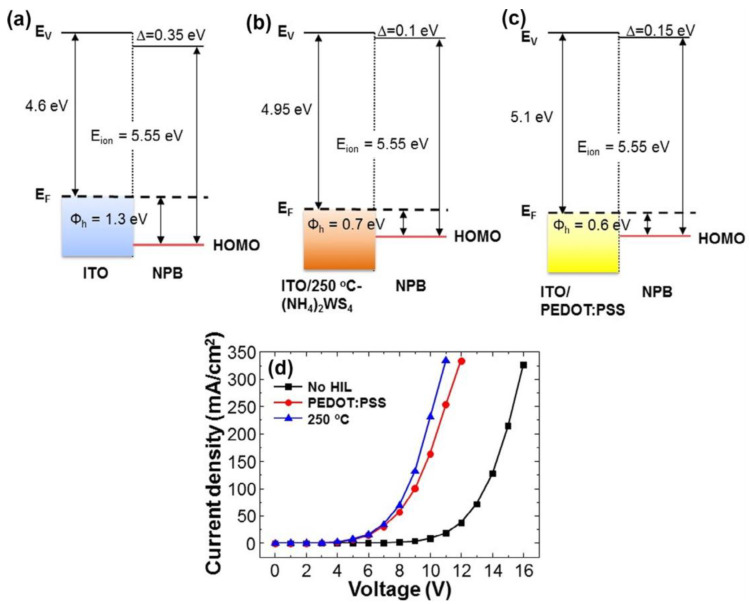
Schematic band diagram of (**a**) ITO/NPB, (**b**) ITO/(NH_4_)_2_WS_4_ annealed at 250 °C, and (**c**) ITO/PEDOT:PSS, (**d**) The current density–voltage characteristics of the hole-only device without HIL, with PEDOT:PSS, and with the 250 °C annealed (NH_4_)_2_WS_4_.(E_F_: Fermi level, Φ_h_: hole injection barrier, HOMO: highest occupied molecular orbital). Reproduced with permission [38]. Copyright 2015, Elsevier B.V.

**Figure 7:**
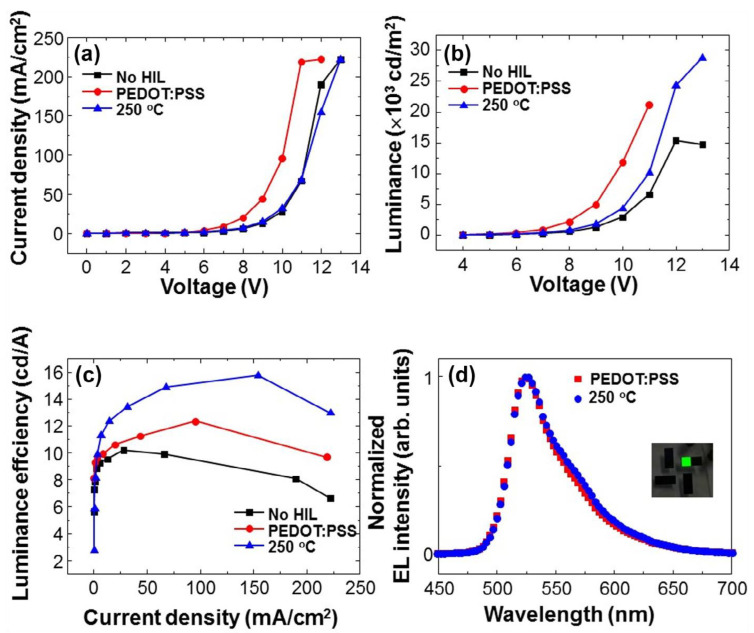
(**a**) Current density–voltage, (**b**) luminance–voltage, (**c**) luminance efficiency–current density, and (**d**) Normalized electroluminescence spectra of the PEDOT: PSS- and 250 °C annealed-(NH_4_)_2_WS_4_-based OLEDs. Reproduced with permission [38]. Copyright 2015, Elsevier B.V.

Two-dimensional (2D) materials such as transition metal dichalcogenides (TMDs) and graphene have garnered significant attention. TMDs, such as MoS_2_, WS_2_, TaS_2_, and TiS_2,_ have been used in field-effect transistors, solar cells, and catalysis, due to their excellent carrier transport properties [35]. WS_x_ and MoS_x_ nanodots were synthesized and used as hole injection layers by Quyet Van Le et al. [36]. The synthesized MoS_2_ nanosheets were composed of two layers with suitable surface WF and superior film morphology [37]. In the research of Quyet Van Le et al., the introduction of 250 °C annealed (NH_4_)_2_WS_4_ between ITO and NPB led to a reduction in Φ_h_ at the ITO/NPB interface from 1.3 V to 0.7 eV, surpassing the PEDOT:PSS/NPB interface by 0.1 eV. Detailed representations of energy level alignment diagrams are depicted in Figure 6a–c, and Figure 7 shows the characteristics of OLEDs compared with the results. The lower Φ_h_ does not cause the higher OLED performance. From the J–V characteristics shown in Figure 7d, at the same applied voltage, the hole injection current density based on 250 °C annealed (NH_4_)_2_WS_4_ is found to be higher compared to that based on PEDOT:PSS. The higher hole injection may lead to a carrier balance as well as balanced electron hole pairs and well-matched energy levels, resulting in better performance of OLEDs based on 250 °C annealed (NH_4_)_2_WS_4_ layers [38].

Phosphomolybdic acid (PMA), a heteropoly acid containing MoO_3_ units, was also used as an HIL for OLEDs due to its physical and optical properties. Compared with PEDOT:PSS-based devices, lower driving voltage was demonstrated. It is worth mentioning that the annealing temperature and atmosphere will have a significant impact on the WF of PMA films, thus affecting the hole injection ability, resulting in different OLED performances [39,40]. 

Graphene oxide (GO), because of its high WF and surface coverage, was synthesized by a tip ultrasonic-assisted liquid-phase exfoliation method with a size distribution of 200–400 nm and used as an HIL for OLEDs [35]. The small-sized GO can introduce more oxygen-containing functional groups due to increasing total edge, leading to a higher WF. When used as an HIL in OLEDs, the carrier injection can be balanced by adjusting the thickness of the GO HIL, thus improving the device performance [41]. Reduced graphene oxide (RGO) HIL can be obtained through chemical and thermal treatment to GO, which exhibits high mechanical strength, high thermal conductivity, electrical performance, and electrochemical activity [42,43,44,45]. 

Perovskites, often used as active materials for LEDs and solar cells, have been used as HILs due to their electronic properties. As we know, if the contact/adhesion between interfaces is poor, the effective migration of holes from the ITO anode to the adjacent organic layer will be hindered [46]. Thus, the better contacts of perovskite at the ITO/HIL and HIL/HTL interfaces are beneficial for hole injection and transmission. A series of different-thickness CH_3_NH_3_PbI_3_ films was prepared on ITO substrates by spin-coating. Maximum brightness of 19,110 cd m^−2^ and EL efficiency of 3.242 cd A^−1^ were obtained for the device with a 22 nm thick HIL. At the same time, the operation stability of OLEDs has been significantly improved. All these improvements can be ascribed to a better balance of charge recombination in the OLED light-emitting zone and probably due to the non-aqueous treatment of the HILs, which increases the chemical stability of the contact surfaces of ITO and CH_3_NH_3_PbI_3_ [2]. 

**Figure 8:**
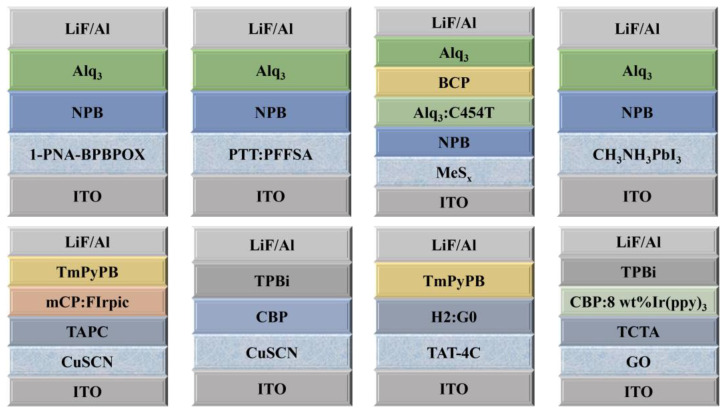
The structures of OLEDs with inorganic HILs.

Inorganic materials have shown great prospects in optoelectronic devices due to favorable thermal stability, high charge carrier mobility, and suitable energy levels. It is unfortunate that a relatively high temperature is needed to anneal the precursor film to fabricate the inorganic HILs, which is detrimental in flexible optoelectronics devices [2]. The structures of OLEDs with inorganic HILs are shown in Figure 8, and the optical properties of OLEDs based on inorganic hole injection materials are shown in Table 3.

## 3. Doped Hole Injection Layer

To overcome the obstacles like limited charge carrier concentration or insufficient charge balance in the device due to large hole injection barriers, doping HILs is a proven technique [51]. Doped materials can be roughly classified into three categories: polymer materials, transition metal oxide materials, metal and metal compound materials.

### 3.1. Polymer Materials

In order to enhance the hole injection, electron blocking, and reduce the massive exciton quenching that arises by using PEDOT:PSS as HILs, different materials are doped with PEDOT:PSS [52,53,54]. By adjusting the proportion of PEDOT and poly(3-hexylthiophene) (P3ht) monomer polymerization, Keon-Soo Jang et al. obtained a composite film with controllable band gap and used it as an HIL in OLEDs [55]. Changyeon Lee et al. prepared HILs with corrugated structures by doping PEDOT:PSS with a polystyrene nanosphere (PS NS). The enhancement of light extraction caused by the corrugated structure may be the main factor improving the efficiency of the devices [56]. Tae-Hee Han et al. doped a tetrafluoroethylene-perfluoro-3,6-dioxa-4-methyl-7octene-sulfonic acid copolymer (PFI) to PEDOT:PSS and used it as an HIL (called GraHIL). The self-organized GraHIL has excellent hole injection/electron-blocking capability because of the gradient WF constructed by the self-organization of PFI, which hindered the exciton quenching at the HIL/EML interface. The self-assembled monolayers can also increase the surface contact angle, decrease root-mean-square (RMS) surface roughness, and improve WF [57]. Similarly, Cheng-Chieh Lo et al. enhanced the performance of OLEDs with acetone and deionized water-modified PEDOT:PSS as HILs. The former exhibited a 34% reduction in surface hydrophobicity and a 200% increase in conductivity. Moreover, the modified HIL showed high transmittances and low refractive indices [58]. 

Except for the PEDOT:PSS polymer, there are some other polymers, which can be used as HILs. WenFen Su et al. balanced the charge injection using a poly (fluorene-co-triphenylamine) (PFO-TPA) crosslinked HIL, and the maximum brightness efficiency of the device was six-times higher than without the PFO-TPA layer [59]. Afsoon Fallahi et al. used Go-doped Poly[(2,5-bis(2-(N,N-diethylammonium bromide)ethoxy)-1,4phenylene)-alt-1,4-phenylene] (PPPNEt_2_.HBr) as an HIL to reduce the hole injection barrier between ITO and HTL, thereby reducing the driving voltage of OLEDs [60].

As indicated in previous studies, surface roughness plays a significant role in device efficiency [61,62]. In 2011, Anna De Girolamo Del Mauro et al. used pure polyaniline (PANI)-poly(styrene sulfonate) (PSS) and dimethyl sulfoxide (DMSO)-doped PANI-PSS as HILs for OLEDs, respectively. The former exhibited a smoother film and, thus, affected the performance of corresponding OLEDs [63]. Mi-Ri Choi et al. mentioned that the doping of a perfluorinated ionomer (PFI) in conductive polymer compositions could enhance the hydrophobicity and WF. Therefore, a solution-processable HIL with a high WF composed of PANI:PSS and PFI (PANI: PSS/PFI) was introduced, which improved the hole injection efficiency and current efficiency of OLEDs. The results showed that high concentrations of PFI play a decisive role in the increase in WF. In addition, when perfluorinated ionic polymers were preferentially located at a film surface, they significantly increased the surface ionization potential [64]. 

### 3.2. Transition Metal Oxide Materials

As mentioned above, the advantages of transition metal oxides can not only be used as hole injection layer alone but also be co-doped with other materials. However, due to the poor solubility of MPcs and transition metal oxides, the fabrication of a thin film typically requires high-vacuum machining and complex operations, resulting in higher production costs [2]. Metal phthalocyanines (MPcs), one of the well-recognized HIL materials in OLEDs, have excellent thermal/chemical stabilities and electronic properties [65]. In 2012, Linsen Li et al. reported a green OLED with MoO_3_-doped phthalocyanine (CuPc) as an HIL, because of the complex of electrical charge transfer between MoO_3_ and CuPc, and the devices showed a turn-on voltage of 4.4 V and PE of 4.3 lm W^−1^ at a brightness of 100 cd m^−2^. The issue of CuPc raising the device turn-on voltage is solved by the doping of MoO_3_ [66]. Different concentrations of transition metal oxides doped with PEDOT:PSS as HILs will affect the OLED performance due to the changed WF [67]. 

Tungsten oxide nanocrystals (WO_3_NCs) were introduced into PANI:PSS by a solution-processed method and used as a hybrid HIL in OLEDs. The existence of PANI:PSS reduced the surface defects and enhanced the film formation of WO_3_NCs efficiently without impacting the conductivity of the OLED. WO_3_NCs improved the WF of the HIL, so that the energy level matched well between the HIL and the anode [68].

In 2018, Weiwei Deng et al. reported an OLED with solution-processed WO_3_-P_2_O_5_, which showed a lower driving voltage for 2.6 V and maximum brightness for 13,553 cd m^−2^, respectively [62]. In 2019, Mangey Ram Nagar et al. reported that a solution-processed OLED with MoO_3_:WO_3_ showed a PE of 22.9 lm W^−1^ at 1000 cd m^−2^, which was ascribed to the robust hole transporting ability, balanced charge carrier, and non-acidic nature of transition metal oxide HILs [69].

### 3.3. Metal and Metal Compound Materials

Active metal materials with low ionization energy are a natural n-type dopant. They are often used as an electron transport material to improve the electron transport capability of devices. However, recent studies have found that the hole injection layer can be doped with metal materials to improve the hole injection capacity.

For example, NiO_x_ has attracted more and more interest in OLEDs due to its good stability, deep valence band, and large band gap. However, the conductivity of a single NiO_x_ film is relatively low, and the energy level mismatch between the ITO and HTL interface hinders the hole injection in OLEDs [70,71]. The doping process is an efficient method to improve the conductivity and carrier mobility of NiO_x_ [72,73]. Cu, Ag, and Li have been used as dopants for NiO_x_ films. This has shown that Cu and Ag can improve the conductivity of NiO_x_ films, and the synergistic effect can enhance the hole injection and improve the efficiency of Li:NiO_x_-based OLEDs [9,74,75]. 

Meiling Shan et al. proved that in CuI-doped m-MTDATA layers, the free carriers generated by charge transfer between CuI and m-MTDATA molecules lead to increased conductivity and form ohmic contact at the ITO/HIL interface [76].

Metal nanoparticles (MNPs) developed localized surface-plasmon resonance (LSPR) effects at the metal–dielectric interface and formed a bi-electrical layer on the anode, which can optimize the Fermi level and improve the hole injection effectively [54,77,78]. The introduction of MNPs, nanorods, nanowire arrays, and nanoclusters as HILs improved the performance of OLEDs [79,80,81,82,83]. In 2018, Jayaraman Jayabharathi et al. used the size-controlled Au-Ag NPs-PEDOT:PSS as an HIL, which has high IQE and light out-coupling efficiency, synergistically enhancing OLED performances. Compared with PEDOT:PSS-based devices, the PE, CE, and EQE of Au-Ag NPs co-doped green OLEDs were increased by 67.3%, 96.1%, and 96.0%, respectively [84]. Similarly, the improved photoelectric performance of GO/Au NP-based OLEDs can be attributed to the LSPR excited by the Au NPs, which cause an enhanced emission intensity and effective energy transfer [85]. GO not only helped to balance the hole and electron injection but optimized the energy level structure of the device, further enhancing the OLED performance [86]. 

Hong Lian et al. prepared 0.5 wt% Fe_3_O_4_@Au, 0.5 wt% Fe_3_O_4_@G, or 0.25 wt% Fe_3_O_4_@SiO_2_ NPs in mixed solutions. By introducing Au into Fe_3_O_4_ NPs, some emerging synergistic effects as well as magnetic effects can be obtained. The combination of light scattering, LSPR, and magnetism effects improved the electroluminescence efficiency of OLEDs. Compared with PEDOT:PSS-based devices, OLEDs with optimal Fe_3_O_4_@Au/PEDOT:PSS and Fe_3_O_4_@G/PEDOT:PSS composite HILs achieved enhancements of 35% in CE and with Fe_3_O_4_@SiO_2,_ NP-PEDOT:PSS HIL obtained a 26.8% increase in CE [87]. Dandan Zhang et al. also mentioned that the Au coating can efficiently stabilize high-moment magnetic NPs. By utilizing the Fe_3_O_4_@Au NPs (FOA NPs), the CE of Alq_3_-based fluorescent OLEDs obtained a 90% increase compared with that without NPs. Additionally, when an external magnetic field (EMF) of 0.5 T was applied, CE can be further increased by approximately 20% due to increased singlet exciton induced by the spin-injected holes via FOA NPs. However, the CE of Ir(ppy)_2(_acac)-based OLEDs decreased mildly. It indicated that the magnetic field effects depend on the system and mechanism of emitting material [88].

## 4. Composite Hole Injection Layer Structure

Apart from the type of material, different device structures also affect the OLED performance. So-called composite HIL refers to two or more layers of film acting as HILs together. The performance of OLED devices is improved through the synergistic action between the layers. And the energy level barrier between different layers is small, which is convenient for carrier injection and, thus, improves device efficiency. The advantages of composite hole injection layers are obvious. The composite HIL favors a continuous power function gradient as well as the formation of ohmic contacts to decrease the injection barrier, thus increasing the hole injection capability and device performance.

These structures can intuitively display the above theories, such as the devices with hole injection layers of Ag_2_O/MoO_x_, MoO_3_/NPB/MoO_3_/CBP, as even the concentration gradient provided a stepped energy level, which greatly facilitated hole injection and, hence, enhanced the injection current [89,90,91]. It has been reported that the presence of charge transfer between MoO_3_ and NPB is also conductive to hole injection [92,93]. 

Yoon Ho Huh et al. proved that GO/MoO_3_/PEDOT:PSS three-layer stacked HIL has better hole injection characteristics than pure PEDOT:PSS [94]. Ling Chen et al. prepared WO_3_/PEDOT:PSS double-layer HILs. The double-layer structure provides a stepped energy level arrangement and smoother surface. The prepared device has an EQE of 12.47%, and a lifetime of 12,551 h was obtained without decreasing efficiency [95]. Lin Lu et al. demonstrated that OLEDs based on F4-TCNQ doping 4,4′4″-Tris(N-(2-naphthyl)-N-phenyl-amino)-triphenylamine(2T-NATA)/2T-NATA composite HILs had lower operating voltages and longer device lifetimes than single-layer devices [96].

**Figure 9:**
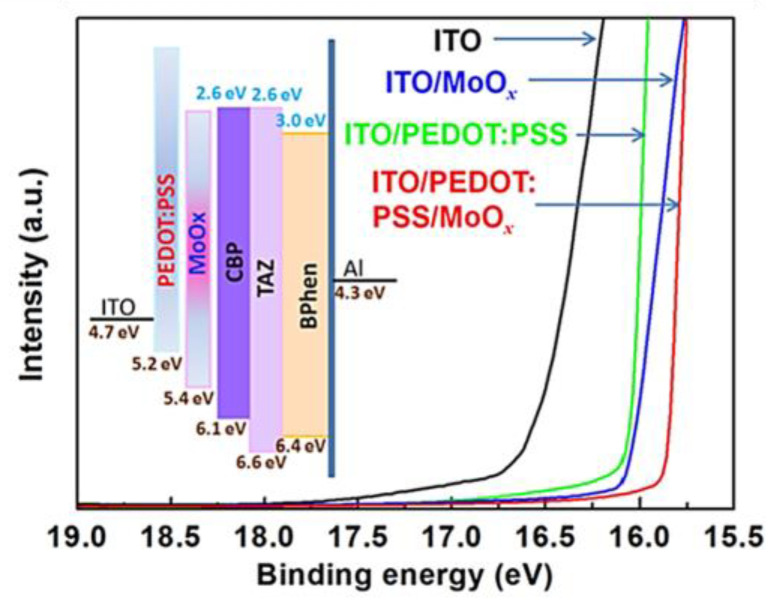
UPS spectra cut-off regions of ITO, ITO/MoO_x_, ITO/PEDOT: PSS and ITO/PEDOT: PSS/MoO_x_. Inset: energy level diagram of UV OLED with PEDOT: PSS/MoO_x_ bilayer HIL. Reproduced with permission [97]. Copyright 2017, American Institute of Physics.

Xiaowen Zhang et al. used a PEDOT:PSS/MoO_x_ bilayer as an HIL for an ultraviolet OLED. The device based on a bilayer HIL exhibited a maximum EQE of 4.6% at 7.28 mA cm^−2^, an improvement of 59% and 31%, compared to single HILs of MoO_x_ or PEDOT:PSS devices, respectively. This can be ascribed to the variation in HIL WF. The schematic inserted in Figure 9 displays an energy level diagram drawn from the measurements of the UPS. The stepped energy level arrangement makes hole injection easier and, correspondingly, promotes the EQE of devices [97].

Except for using a double-hole injection layer, the synergy between the hole injection layer and other layers can also be used. For example, although the roles of HILs and hole blocking layers were opposite, maximum luminance efficiency and PE could be obtained when their thicknesses were in a specific combination [98].

## 5. Preparation Methods

**Figure 10:**
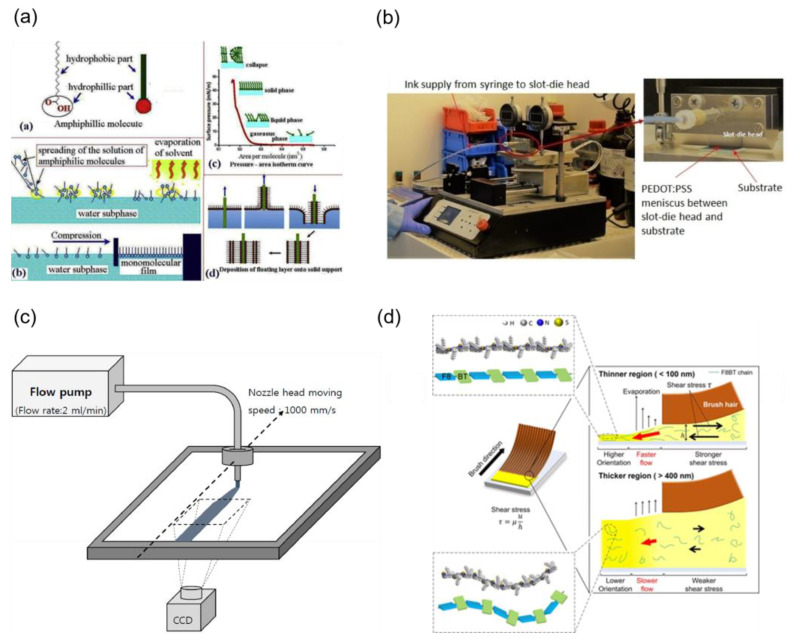
(**a**) LB film technology, (**b**) slot-die coating, (**c**) nozzle printer, (**d**) brush-coating. Reproduced under terms of the CC-BY license [100,101,102]. Copyright 2018, The Authors, published by Elsevier Ltd.; Copyright 2019, The Authors, published by MDPI and Copyright 2018, The Authors, published by MDPI. Reproduced with permission [103]. Copyright 2020, American Chemical Society.

At present, the most commonly used methods for preparing hole injection layers are spin-coating and vacuum deposition. Spin-coating has been widely implemented in the fabrication of polymer-based OLEDs due to its cost-effective and easy-to-manufacture nature, but it suffers from low material utilization and difficulties in large-scale industrial production [23,99]. The HIL prepared by vacuum evaporation has the advantages of high purity and density, unique structure, and properties in the film. However, vacuum processing is costly and requires complicated manufacturing equipment [23]. As alternatives, some methods have been developed, like spray-coating, roll-to-roll coating, screen-printing, blade-coating, slot-die coating, Langmuir–Blodgett (LB) deposition technique, nozzle printing technology, and brush coating [99]. Figure 10 shows a schematic diagram of different preparation methods.

LB technology can control the film arrangement and thickness evenly, and the preparation process does not require high temperature and pressure. The arrangement features of carbon sheets lead to significant changes in electrical ability, which greatly influences the performance of OLED devices. The LB deposition technique is superior in inhibiting the wrinkling and folding of GO sheets substantially. The higher surface pressure makes the reduced graphene oxide (RGO) sheets’ arrangement more compact and continuous, forming compact and uniform conductive paths for efficient carrier transport. Yajie Yang et al. used LB technology to incorporate orderly and controllable-thickness RGO flakes between the ITO and HIL, which served to planarize the anode surface. The maximum brightness of the device was about 6232 cd m^−2^, which was more than the spin-coating RGO devices [104]. However, LB technology is limited in terms of expensive equipment and scarce available materials.

Aqueous polymer-based HILs are usually made by large-area slot-die coating due to the low material waste. Large-area OLEDs for illumination were fabricated by Kwang-Jun Choi using slot-die coating. The performance of OLEDs with optimized slot-die-coated multiple layers is comparable to the performance of OLEDs with spin-coated HIL or vacuum-evaporated HTL [105]. Moreover, Amruth C et al. obtained uniform PEDOT:PSS films by modifying the parameters of slot-die processing, such as the speed of coating, ink flow rate, and substrate temperature [101]. This method can also be used to prepare flexible devices [106]. However, it is tedious to explore suitable parameters in the slot-die technique for different materials, which is more suitable for large-scale production.

For fear of the influence of the high surface tension of water, a PEDOT:PSS solution mixed with various concentrations of ethanol (EtOH) was employed as HILs by using the nozzle printing technique. The CE of the device containing 90% ethanol solution reached 27 cd A^−1^ [102]. In 2021, Kwon-Yong Shin et al. successfully deposited PEDOT:PSS solution diluted 90 vol% with EtOH as HIL by employing a micro-multi-nozzle spray-coating method [107]. Despite the fact that the HIL preparation based on printing technology has numerous benefits, like simple preparation, low cost, and suitability for large-area processing, the uniformity of the prepared films is still a critical issue. 

The brush-coating technique has a material utilization rate close to 100% and has no material constraints. In 2021, red, green, blue (RGB), and white phosphorescent OLEDs were manufactured by sequential brush coating on smooth HILs. The CE of the brush-coated white OLED device was comparable to that of the spin-coated device, which was about 29.3 cd A^−1^. Furthermore, the peak current efficiencies of the brush-coated red, green, and blue phosphorescent OLEDs were 16.7, 30.4, and 20.9 cd A^−1^, respectively [99].

It is requisite to select appropriate film preparation methods according to the materials of the functional layer and substrate.

## 6. Different Treatment Methods

The prepared hole injection layer will be affected by atmosphere, temperature, humidity, and even solar radiation, thus affecting the device performance [84]. Therefore, people try to use different post-treatment methods for hole injection layer to improve the device performance.

Ultraviolet/ozone (UVO) treatment is proved to be an efficient method for HILs to increase the WF, smooth the defects of films, and improve the hole injection [57,108,109]. WS_x_ and MoS_x_ nanodots were synthesized and used as hole injection layers by Quyet Van Le et al. After the UVO treatment, the WF of the nanodots was raised from 4.3–4.4 to 5.0–5.1 eV. The maximum luminance efficiency of the UVO-MoS_x_ based OLED was 14.7 cd A^−1^, while that of PEDOT:PSS-based devices was 13.1 cd A^−1^. Moreover, the use of UVO-treated MoS_x_ or WS_x_ nanodots as HILs could prolong the stability of OLEDs in air [36]. 

HILs prepared on a substrate with plasma treatment such as CF_4_ and Ar can also improve device performance [110,111]. In a report by Joo Hyung Kim et al., the optimized Ar plasma treatment can improve the physical characteristics of WO_3_ HIL and, thus, enhance the device performance [111].

The influence of temperature and atmosphere on the preparation of the hole injection layer cannot be ignored. Sul A. Choi et al. discovered that the interfacial morphology as well as the phase state were altered by the substrate temperature, which had an impact on the copper phthalocyanine (CuPc) OLED performance. As the substrate temperature elevated from room temperature to 300 °C, the crystal structure of CuPc films changed from an orthorhombic crystal α-phase to a monoclinic crystal β-phase. The α-phase favors hole transport, and the β-phase increases the surface roughness. Consequently, 10 nm of β-phase CuPc was added on top of the α-phase CuPc layer, which was used as an HIL. This enhanced both the CuPc carrier migration and the device brightness in comparison to the pure β-phase CuPc-based OLEDs [112]. Also, the structure and conductivity of GO were altered by thermal treatment, thus forming RGO with better conductivity [104].

Furthermore, optimizing the carrier injection could employ the polar solvent vapor annealing (PSVA) method, resulting in a significant reduction in charge accumulation at the OLED interface. After the double-layer PSVA treatment, the efficiency roll-off of OLEDs dropped from 33.3% to 26.6% [113].

Light treatment of materials before preparing hole injection layers will also change the performance of devices. Janardan Dagar et al. demonstrated that the device properties with 2D MoO_3_ nano-flakes as the HIL are closely related to the irradiation time. This is attributed to the transformation of 2D MoO_3_ nano-flakes from a semiconductor phase to a metal phase after increasing the irradiation time [114]. Hsi-Kang Shih et al. used PTC-U irradiated with UV for 1 h as the hole injection layer, and hole injection, hole transport, and solvent resistance properties of PTC-U photo-crosslinked for 1 h showed a great improvement, as compared with the PTC-U without UV irradiation [22].

## 7. Conclusions and Outlook

Though OLED televisions and panels are marketized now, there is still room for growth. The relatively lower efficiency of blue OLEDs and the severe efficiency roll-off need to be perfected. The introduction of optimized HILs between electrodes and HTL is a promising solution to facilitate charge injections into EML and prevent the excitons from being quenched by electrodes at the same time. 

In this review, we described the materials from 2010 to 2022 that can be used as hole injection layers and classified them into organic materials and inorganic materials. It is clear that organic materials reduce the cost of preparation, but improvements in the efficiency of OLEDs are not as good as for inorganic materials. Moreover, a method of improving device performance by doping two or more materials is described. The roughness and WF of the device surface can be changed by doping. Certain materials can form self-organizing surfaces to reduce exciton quenching. The LSPR effects of mental nanoparticles and the applied magnetic field can also improve the device performance. In addition, the methods used to prepare the hole injection layer are described, such as spin-coating, brush coating, LB deposition, slot-die coating, nozzle printing, evaporation, etc. The comparison of various methods is also pointed out in this paper. Finally, we described the influence of the external environment such as temperature and sunlight on the hole injection layer film.

In short, the performance of OLEDs was enhanced by introducing appropriate HILs. The optimized HIL can provide better energy level matching, reducing the negative influence between adjacent layers, balancing the injection of carriers, and leading to the enhanced optoelectronic properties of OLEDs. However, there are still many pending problems. Different types of composite hole injection layers are waiting to be excavated, as well as whether there are other mechanisms to choose, except forming ladder potential barriers. In addition to the existing hole injection layer preparation methods, there is a demand for the development of more energy-saving, cost-saving, and simpler methods. The implementation of low-cost, large-scale manufacturing will certainly be accelerated because of further awareness and developments.

## Figures and Tables

**Table 1:** The optical properties of the devices with biomaterial HILs.

Commercial Symbol	Work Function [eV]	CE [cd A^−1^]	PE [lm W^−1^]	EQE [%]	Turn-On Voltage [V]	Maximum Brightness [cd m^−2^]	Ref.
LR-PU	-	-	1.24	0.37	3.7	6310	[20]
DNA	-	-	-	-	4.25	45,000	[19]
PTC-U	-	-	3.8	2.3	3.5	43,652	[21]
PTC-A	-	-	4.6	2.4	3	47,226	[21]

**Table 2:** The optical properties of OLEDs based on transition metal oxide hole injection materials.

Commercial Symbol	Work Function [eV]	CE [cd A^−1^]	PE [lm W^−1^]	EQE [%]	Turn-On Voltage [V]	Maximum Brightness [cd m^−2^]	Ref.
MoO_3_	-	-	-	-	2.4	140,000	[32]
V_2_O_5_	5.6	65	35	-	5.8	153,600	[23]
WO_3_	5.6			12.7	-	-	[34]
MoO_3_	5.67	32.6	25.1	13.7	-	-	[34]
V_2_O_5_	5.8	-	-	11	-	-	[34]
MoO_x_	-	-	5	-	5.2	-	[33]

**Table 3:** The optical properties of OLEDs based on inorganic hole injection materials.

Commercial Symbol	Work Function (eV)	CE (cd A^−1^)	PE (lm W^−1^)	EQE (%)	Turn-On Voltage (V)	Maximum Brightness (cd m^−2^)	Ref.
1-PNA-BPBPOX	4.86	-	2.8	-	5.7	-	[47]
PTT: PFFSA	-	-	-	-	-	-	[14]
MeSx	5.1	-	-	-	4	23,300	[36]
CH_3_NH_3_PbI_3_	-	-	3.210	-	2.5	19,110	[2]
CuSCN	-	17.6	5.6	9.2	5.6	26,424	[48]
CuSCN	-	53.7	40.3	13.9	4.2	15,180	[49]
TAT-4C	-	-	39.2	11	2.7	-	[50]
GO	5.16	73.14	53.95	20.63	3	131,600	[41]
